# Reshaping the path of mild cognitive impairment by refining exercise prescription: a study protocol of a randomized controlled trial to understand the “what,” “for whom,” and “how” of exercise to promote cognitive function

**DOI:** 10.1186/s13063-022-06699-7

**Published:** 2022-09-09

**Authors:** Cindy K. Barha, Ryan S. Falck, John R. Best, Lindsay S. Nagamatsu, Ging-Yuek Robin Hsiung, A. William Sheel, Chun Liang Hsu, Arthur F. Kramer, Michelle W. Voss, Kirk I. Erickson, Jennifer C. Davis, J. Kevin Shoemaker, Lara Boyd, Rachel A. Crockett, Lisanne ten Brinke, Louis Bherer, Joel Singer, Liisa A. M. Galea, Claudia Jacova, Alexis Bullock, Sofia Grant, Teresa Liu-Ambrose

**Affiliations:** 1 Department of Physical Therapy, University of British Columbia (UBC), Vancouver, British Columbia Canada; 2 Djavad Mowafaghian Centre for Brain Health, UBC, Vancouver, British Columbia Canada; 3 Centre for Hip Health and Mobility, Vancouver Coastal Health Research Institute, Vancouver, British Columbia Canada; 4 Gerontology Research Centre, Simon Fraser University, Vancouver, British Columbia Canada; 5 Department of Gerontology, Simon Fraser University, Vancouver, British Columbia Canada; 6 Department of Psychiatry, UBC, Vancouver, British Columbia Canada; 7 Faculty of Health Sciences, School of Kinesiology, Western University, London, Ontario Canada; 8 Brain and Mind Institute, Western University, London, Ontario Canada; 9 Division of Neurology, UBC, Vancouver, British Columbia Canada; 10 School of Kinesiology, UBC, Vancouver, British Columbia Canada; 11 Hinda and Arthur Marcus Institute for Aging Research, Hebrew SeniorLife, Boston, MA USA; 12 Department of Psychology, Northeastern University, Boston, MA USA; 13 Beckman Institute, University of Illinois, Urbana, IL USA; 14 Department of Psychological and Brain Sciences, University of Iowa, Iowa City, IA USA; 15 Iowa Neuroscience Institute, University of Iowa, IA Iowa City, USA; 16 Department of Psychology, University of Pittsburgh, Pittsburgh, PA USA; 17 Center for the Neural Basis of Cognition, University of Pittsburgh, Pittsburgh, PA USA; 18 Neuroscience Research Institute, AdventHealth, Orlando, FL USA; 19 Social and Economic Change Laboratory, Faculty of Management, UBC–Okanagan, Kelowna, Canada; 20 Department of Medicine, University of Montreal, Montreal, Quebec Canada; 21 Research Centre, Montreal Heart Institute, Montreal, Quebec Canada; 22 Research Center, Institut Universitaire de Geriatrie de Montréal, Montreal, Quebec Canada; 23 School of Population and Public Health, UBC, Vancouver, British Columbia Canada; 24 Providence Healthcare Research Institute, Centre for Health Evaluation and Outcome Sciences, Vancouver, British Columbia Canada; 25 Department of Psychology, UBC, Vancouver, British Columbia Canada; 26 School of Graduate Psychology, Pacific University, Hillsboro, OR USA

**Keywords:** Randomized controlled trial, Mild cognitive impairment, Aerobic training, Resistance training, Cognition, Mobility, Biomarkers, Exercise

## Abstract

**Background:**

Targeted exercise training is a promising strategy for promoting cognitive function and preventing dementia in older age. Despite the utility of exercise as an intervention, variation still exists in exercise-induced cognitive gains and questions remain regarding the type of training (i.e., what), as well as moderators (i.e., for whom) and mechanisms (i.e., how) of benefit. Both aerobic training (AT) and resistance training (RT) enhance cognitive function in older adults without cognitive impairment; however, the vast majority of trials have focused exclusively on AT. Thus, more research is needed on RT, as well as on the combination of AT and RT, in older adults with mild cognitive impairment (MCI), a prodromal stage of dementia. Therefore, we aim to conduct a 6-month, 2 × 2 factorial randomized controlled trial in older adults with MCI to assess the individual effects of AT and RT, and the combined effect of AT and RT on cognitive function and to determine the possible underlying biological mechanisms.

**Methods:**

Two hundred and sixteen community-dwelling adults, aged 65 to 85 years, with MCI from metropolitan Vancouver will be recruited to participate in this study. Randomization will be stratified by biological sex and participants will be randomly allocated to one of the four experimental groups: (1) 4×/week balance and tone (BAT; i.e., active control); (2) combined 2×/week AT + 2×/week RT; (3) 2×/week AT + 2×/week BAT; or (4) 2×/week RT + 2×/week BAT. The primary outcome is cognitive function as measured by the Alzheimer’s Disease Assessment Scale-Cognitive-Plus. Secondary outcomes include cognitive function, health-related quality of life, physical function, actigraphy measures, questionnaires, and falls. Outcomes will be measured at baseline, 6 months (i.e., trial completion), and 18 months (i.e., 12-month follow-up).

**Discussion:**

Establishing the efficacy of different types and combinations of exercise training to minimize cognitive decline will advance our ability to prescribe exercise as “medicine” to treat MCI and delay the onset and progression of dementia. This trial is extremely timely as cognitive impairment and dementia pose a growing threat to global public health.

**Trial registration:**

ClinicalTrials.gov NCT02737878. Registered on April 14, 2016.

## Introduction

Dementia is one of the most pressing health care issues of the twenty-first century. Individuals with cognitive impairment and dementia have reduced quality of life as they lose their functional independence [1]. As the proportion of the population over 65 years continues to increase, dementia will place increasing demands and costs on the public health system [2]. Thus, the societal value of identifying and developing effective intervention and prevention strategies cannot be overstated [3]. If the onset and progression of dementia were delayed by 1 year, there would be 9 million fewer cases by 2050 [3].

Effective pharmacologic treatment of dementia remains a major challenge [4]. Exercise is a promising strategy for preventing dementia [5]. Notably, exercise significantly reduces key cardiometabolic risk factors for dementia [6, 7], such as hypertension and type 2 diabetes, for both Alzheimer’s disease and vascular cognitive impairment—the two most common types of dementia.

Broadly, there are two distinct forms of exercise: (1) aerobic training (AT; e.g., running), aimed at improving cardiovascular health; and (2) resistance training (RT; e.g., lifting weights), aimed at improving muscle strength. Each type of exercise training has its own distinct physiology and benefits [8]. Evidence from randomized controlled trials (RCTs) suggests that both AT and RT enhance cognitive function in older adults without cognitive impairment and dementia [9, 10]. However, questions regarding the type of training (i.e., what), as well as moderators (i.e., for whom) and mechanisms (i.e., how) of benefit still remain [11, 12].

In regard to what type of exercise, the majority of published RCTs have focused solely on AT [9, 13]. Thus, more research is needed on RT, as well as on the combination of AT and RT. Importantly, to our knowledge, there is no single published high-quality RCT that has examined both the individual and combined effects of AT and RT on cognitive function in older adults. Establishing the efficacy of different types and combinations of exercise training will advance our understanding of how to best prescribe exercise to attenuate cognitive decline. For example, exercise prescription could be personalized according to deficits in specific cognitive domains. For those who do not have the mobility to uptake AT, RT could be recommended as an evidence-based alternative.

For whom exercise benefits, there is good evidence that targeted exercise training improves cognitive function in healthy older adults [9, 10, 14–16]. However, the role of exercise interventions among those at increased risk for dementia, such as older adults with mild cognitive impairment (MCI), is not well established [14, 17–21]. Currently, there is wide recognition that MCI represents the prodromal stage of Alzheimer’s disease, vascular cognitive impairment, and mixed dementia [22–25]. MCI is a clinical entity characterized by cognitive decline greater than that expected for an individual’s age and education level but that does not interfere notably with everyday function [26]. Individuals with MCI are at increased risk for dementia [22]; those with MCI develop Alzheimer’s disease at a rate between 10 and 30% annually [27, 28], whereas those without MCI develop dementia at a rate between 1 and 2% annually [27]. Evidence also suggests that older adults with MCI experience significant declines in quality of life [1, 29]. Thus, older adults with MCI represent an ideal target population for intervention strategies, as the preservation of their cognitive function will likely prolong their ability to live independently and with quality.

Evidence suggests older females may reap greater cognitive gains from AT than older males, particularly in the cognitive domain of executive functions [10, 13, 30–32]. Much of this evidence comes from meta-analyses comparing RCTs of AT that have a high percentage of older females to RCTs with low percentage of older females (i.e., data not disaggregated by biological sex) [13, 33]. Direct RCT evidence is restricted to post hoc analyses [30–32], which are not definitive because statistical power is lacking. Moreover, whether there are sex differences in the effect of RT on cognition is largely unexplored.

Our current understanding of how exercise promotes cognitive function in humans is limited and largely focuses on central induction of neurotrophic factor cascades, such as brain-derived neurotrophic factor (BDNF) and insulin-like growth factor (IGF-1). Evidence from rodent models suggests different types of exercise training may promote cognitive health through divergent pathways, with AT preferentially increasing BDNF and RT preferentially increasing IGF-1 [34]. In humans, AT increases peripheral BDNF levels [35, 36] and, while less studied, RT increases IGF-1 [37, 38].

AT and RT may also promote cognitive function through parallel pathways, including reducing peripheral and central inflammation, which is associated with the pathogenesis of MCI [39]. Indeed some evidence supports the ability of AT and RT to reduce circulating levels of certain inflammatory markers in those with MCI [40].

Gaining insights into the underlying biological mechanisms will enable the refinement of existing exercise recommendations. For example, if RT preferentially increases IGF-1 compared with AT, then RT may be the more appropriate recommendation for individuals with the BDNF Val66met polymorphism, a common single-nucleotide polymorphism resulting in reduced activity-dependent secretion of the mature form of BDNF [41].

Thus, we aim to conduct a 2 × 2 factorial RCT of exercise in community-dwelling older adults with MCI. Our primary objective is to assess the individual effects of AT and RT, as well as the combined (i.e., interaction) effect of the two types of exercise training on cognitive function, as measured by the Alzheimer’s Disease Assessment Scale-Cognitive-Plus (ADAS-Cog-Plus). The secondary objective is to assess sex differences. The tertiary objectives are to explore the mechanisms underlying the individual and interaction effects of AT and RT, and to explore the potential moderation of effects by the BDNF Val66Met polymorphism.

## Methods

### Design and setting

**Fig. 1:**
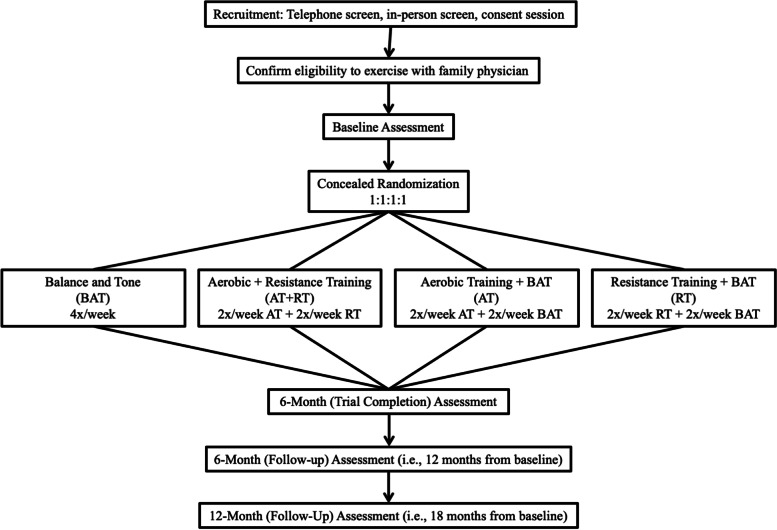
Overview of study design from recruitment to follow-up

We will conduct a single-blinded, superiority, 2×2 factorial-arm RCT in a research center (Vancouver, British Columbia, Canada) with 216 community-dwelling older adults with MCI, as determined by a baseline Montreal Cognitive Assessment (MoCA) score < 26/30, with subjective memory complaints (SMC), without significant functional impairment, and no dementia (Clinical Dementia Rating (CDR) < 1.0) [42, 43], aged 65 to 85 years, living in Metro Vancouver, BC, Canada. This study will include a 6-month intervention and a 12-month follow-up (i.e., 18 months from baseline; Fig. 1). There will be a dedicated research coordinator (non-blinded) and trained assessors (blinded). Standardized protocols will be developed, and study personnel will be trained by the research team. Assessments and exercise sessions will occur at a research laboratory on the Vancouver General Hospital campus, Vancouver, Canada.

### Recruitment

Individuals will be recruited from both the community, with advertisements placed in community centers and newspapers in Greater Vancouver, and from the University of British Columbia Hospital Clinic for Alzheimer’s Disease and Related Disorders. All individuals who receive care at the clinic have the option to sign a consent form providing access to their records for research purposes and indicating their willingness to be approached for research studies. Individuals interested in participating will be screened by telephone to check for eligibility based on our inclusion and exclusion criteria, and the Physical Activity Readiness Questionnaire for Everyone (PAR-Q Plus) [44], a screening measure of physical readiness for exercise. Research staff will invite eligible participants to attend a consent and screening session, either over the phone or in-person, during which details of the study will be provided.

### Time frame

Participant enrollment began on November 13, 2017, and the final assessment is anticipated to be completed by December 2023. The COVID-19 pandemic impacted recruitment for much of 2020. As of May 25, 2022, 176 individuals (81% of the target sample) have been recruited and randomized.

### Eligibility

#### Inclusion criteria

We will include community-dwelling females and males who meet the following inclusion criteria: (1) aged between 65 and 85 years; (2) have subjective memory complaints, defined as the self-reported feeling of worsening memory, as determined by a standard question [45]; (3) have baseline MoCA score of < 26/30 [46]; (4) have a Mini-Mental State Examination (MMSE) score > 22 [47] and a Clinical Dementia Rating score < 1.0 [43]; (5) do not have any significant impairment in daily function as indicated by a score of > 6/8 on the Lawton and Brody Instrumental Activities of Daily Living Scale [48]; (6) score < 5/15 on the Geriatric Depression Scale (GDS) [49]; (7) completed high school education; (8) live in their own home; (9) read, write, and speak English with acceptable visual and auditory acuity; (10) not expected to start, or are stable, on a fixed dose of anti-dementia medications (e.g., donepezil, galantamine); (11) provide a signed and dated informed consent document; (12) able to walk independently without an aid; (13) in sufficient health to participate in the exercise programs, based on their medical history, the PAR-Q Plus [44], and written approval by their family physician (if required); and (14) have the capacity to comply with the scheduled assessments and exercise sessions.

#### Exclusion criteria

We will exclude individuals who are (1) engaged in moderate-intensity aerobic exercise > 60 minutes per week, in the 3 months prior to study entry; (2) engaged in progressive resistance training > 1×/week in the 3 months prior to study entry; (3) diagnosed previously with dementia of any type; (4) clinically suspected to have neurodegenerative disease as the cause of MCI that is not Alzheimer’s disease, vascular cognitive impairment, or both (e.g., multiple sclerosis, Parkinson’s disease, Huntington’s disease, frontotemporal dementia); (5) at high risk for cardiac complications during exercise or unable to self-regulate activity or to understand recommended activity level; (6) diagnosed by their family physician with clinically important peripheral neuropathy or severe musculoskeletal or joint disease that impairs mobility; (7) on a new or recent (i.e., less than 3 months from study entry) or changed dose of medications that may negatively affect cognitive function, such as anticholinergics (i.e., typical and atypical antipsychotics) and anticonvulsants (e.g., gabapentin, valproic acid); (8) on any hormone therapy (estrogen, progesterone, or testosterone) in the last 24 months; or (9) planning to participate, or already enrolled in, a concurrent clinical drug or exercise trial.

A subset (~40%) of right hand-dominant participants will undergo magnetic resonance imaging (MRI) scanning. We will exclude participants who do not meet the specific scanning requirements of the UBC MRI Research Centre or do not provide consent. Specifically, participants will be excluded from the MRI scanning if they have any of the following: pacemaker, brain aneurysm clip, cochlear implant, surgery or tattoos within the past 6 weeks, electrical stimulator for nerves or bones, implanted infusion pump, history of any eye injury involving metal fragments, artificial heart valve, orthopedic hardware, other metallic prostheses, any blood vessel filter, coil or catheter, ear or eye implant, bullets, or other metallic fragments.

### Sample size considerations

**Fig. 2:**
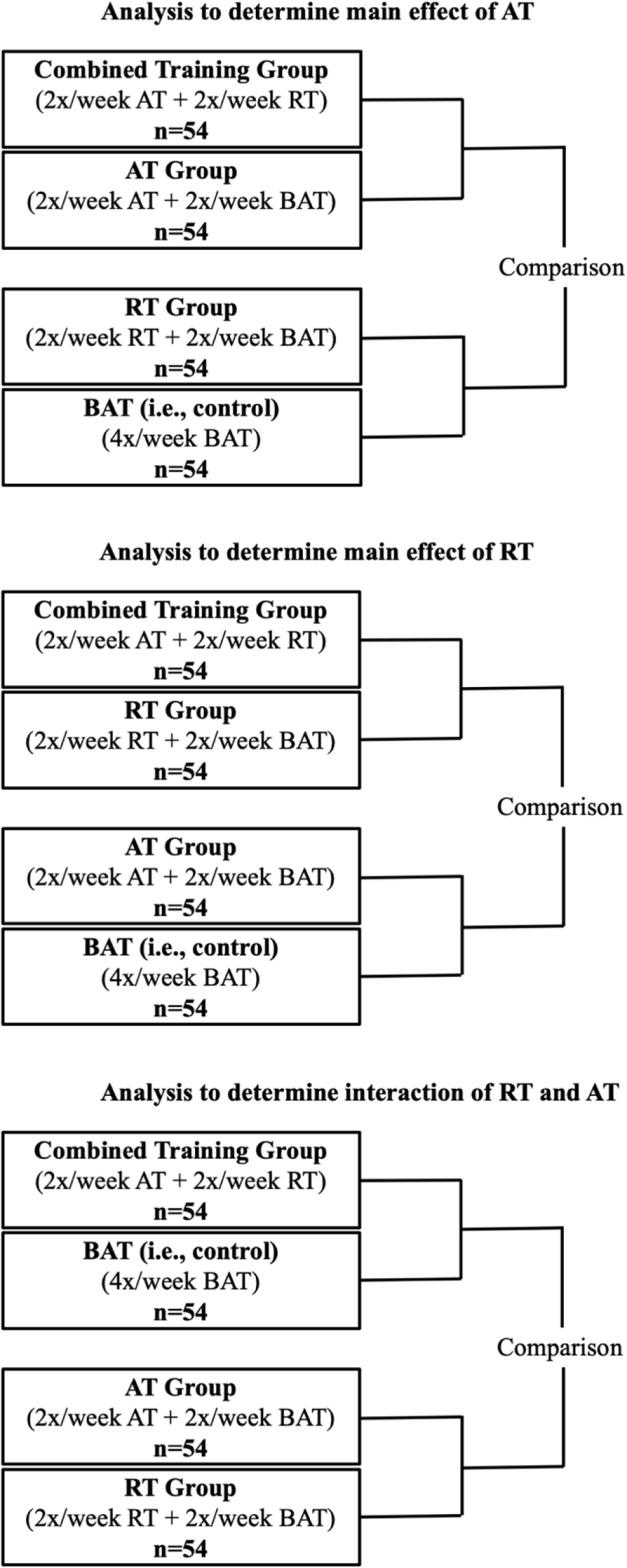
Schematic representation of the three primary comparisons in the factorial randomized controlled trial design

The planned sample size was determined with the aim of testing the main effects of AT and RT on changes in cognitive function, as assessed by the ADAS-Cog-Plus [50]. In computing the required sample size, we used G*Power and the sample analytic model in the statistical analyses. Unpublished data from a separate set of 96 older adults with MCI from our laboratory estimated that the correlation between baseline ADAS-Cog-Plus and the same measure collected 6 months later was approximately 0.80 [51]. This value was used to calculate the assumed residual variance in the analysis of covariance (ANCOVA) model of 0.36 (i.e., 1 − 0.80^2^). With a targeted enrolment of 216 individuals and accounting for an overall dropout of 15% (assumed to impact each intervention arm equally), we will have a power of 0.80 to detect a standardized mean difference (SMD) of 0.27 for the effect of AT or RT on cognitive function with a two-tailed alpha of 0.05 applied to each comparison [9]. The SMD of 0.27 concurs with findings from recent meta-analyses [9, 52, 53]. To detect an interaction between AT and RT with 0.80 power, the true effect size of 0.53 is required. Figure 2 depicts the 3 planned primary comparisons to examine the main and interaction effects of AT and RT.

Our secondary objective is to test whether biological sex moderates the effect of exercise (AT and RT, separately) on changes in cognitive function, as assessed by the ADAS-Cog-Plus. We conducted two meta-analyses [10, 52] and observed an average sex difference, as determined by comparing trials with high versus low proportion of female, in AT (SMD=0.75) and in RT (SMD=0.65) on executive functions and episodic memory. Using these values as estimates of the true effect sizes of the biological sex by exercise interaction effects, a total sample size of 216 (108 men and 108 women) randomly assigned to one of the four conditions will provide greater than 0.90 power with a two-tailed alpha of 0.05 and accounting for an overall dropout rate of 15% (assumed to impact each intervention arm equally).

### Data entry

No personal identifiers will be acquired during data collection. All paper-based data will be stored in locked cabinets and all alphanumeric data will be entered by a trained study personnel who will conduct range checks for data values. All alphanumeric data and neuroimaging will be stored on a secured server hosted by UBC. All data will be deidentified.

### Measurements

**Fig 3:**
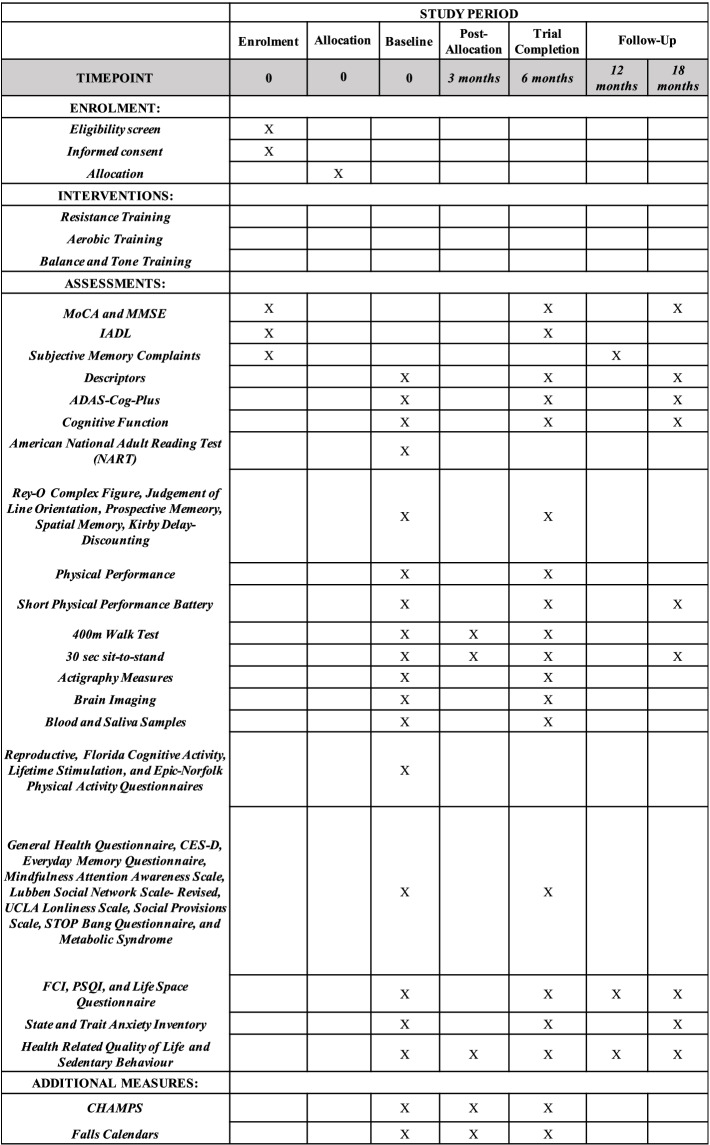
Schedule of enrolment, interventions, and assessments according to the SPIRIT Checklist

There will be three key measurement points: baseline, 6 months (trial completion), and 18 months (i.e., 12-month follow-up). To minimize attrition over the 12-month follow-up, participants will be contacted at 6 months post trial completion to complete a set of questionnaires. Baseline measurements will be obtained prior to randomization. See Fig. 3 for the schedule of all study measures.

#### Consent and screening session

Subjective memory complaints will be determined with one verbal yes/no question: “Do you feel like your memory or thinking is becoming worse?”. Global cognition will be assessed with the MoCA [46] and MMSE [47]. The Lawton and Brody Instrumental Activities of Daily Living Scale and the GDS will also be administered. The Edinburgh Handedness Questionnaire will be administered to assess handedness [54].

#### Descriptors

Standing height in centimeters and mass in kilograms will be ascertained using standardized equipment. General health, demographics, socioeconomic status, and education will be obtained via questionnaire. The Functional Comorbidity Index will estimate the degree of comorbidity associated with physical functioning [55]. The GDS will be used to assess depressive symptoms [49]. The EPIC-Norfolk Physical Activity Questionnaire will measure physical activity levels [56]. Engagement in cognitive stimulating activities will be measured with the Florida Cognitive Activity Questionnaire [57] and the Lifetime Participation in Cognitively Stimulating Activities [58]. The National Adult Reading Test will be used to estimate premorbid intellectual functioning [59]. The Reproductive History Questionnaire will assess reproductive histories of participants [adapted from [60]]. To examine potential genetic moderation of exercise efficacy, we will take a targeted approach for common single-nucleotide polymorphisms that have previously been implicated in cognitive aging and exercise-induced cognitive gains (e.g., BDNF Val66Met polymorphism, APOE4) [11, 61]. DNA will be extracted from whole blood by standard protocol, and genotype will be determined by a TaqMan by-design assay.

#### Primary outcome

Our primary measure will be the ADAS-Cog-Plus [50]. The ADAS-Cog-Plus uses a multidimensional item response theory model to generate a global cognitive score from the 13-item ADAS-Cog [62] and additional standard cognitive assessments. For this trial, we will use the 13-item ADAS-Cog (0-85 points), Trail Making Tests (Parts A and B), Digit Span Forward and Backward, Digit Symbol Substitute Test, animal and vegetable fluency, Rey Auditory Verbal Learning Test, and Clock Drawing test as the input variables into the scoring algorithm. Lower ADAS-Cog-Plus scores represent better cognitive performance; scores range from −1.0 to +1.0 [e.g., −1.0 indicates healthy cognitive functioning, 0.0 indicates MCI, and +1.0 indicates dementia [63]].

#### Secondary outcomes

##### Cognitive function

A battery of computerized and standardized paper and pen neuropsychological tests will be used to assess different cognitive domains. The computerized battery will include the National Institutes of Health Toolbox Cognition Battery [64, 65], a comprehensive neuropsychological battery with normative values. Specifically, we will use its (a) Dimensional Change Card Sort Test to measure set shifting; (b) Flanker Inhibitory Control and Attention Test to measure response inhibition and attention; (c) List Sorting Working Memory Test to measure working memory; and (d) Picture Sequence Memory Test to measure episodic memory. Spatial memory will be measured with a computerized paradigm [66]. The task requires participants to recall the spatial location of one, two, or three dots presented on a computer screen. Reaction time and accuracy will be recorded.

Standardized paper and pen neuropsychological tests will assess executive functions, verbal fluency, visual memory, prospective memory, and visuospatial ability. Specifically, the battery will include the individual components of ADAS-Cog-Plus, such as the 13-item ADAS-Cog, Trail Making Tests (set shifting), Digit Span Forward and Backward (working memory), Digit Symbol Substitution Test (processing speed), animal and vegetable fluency (verbal fluency), Rey Auditory Verbal Learning Test (verbal memory), and Clock Drawing. For the 13-item ADAS-Cog, a change of 3.0 points is a minimally important difference [67]; higher scores on the 13-item ADAS-Cog indicate greater cognitive impairment (range 0–85 points).

In addition, we will administer the Stroop Colour Word Test to assess response inhibition and selective attention [68]. Visual memory will be assessed by the Rey-Osterrieth Complex Figure Test [69]. Prospective memory, the ability to remember and perform an intended action at a specific time in the future, will be assessed by the Prospective Memory Test [70]. We will use the Judgement of Line Orientation to test visuospatial skills [71].

##### Health-related quality of life and wellbeing

We will evaluate health-related quality of life using the EuroQol-5 Domain-5 Level (EQ-5D-5L) [72]. This is a preference-based utility instrument that provides weightings for quality-adjusted life years (QALYs). A QALY is a metric used to assess health-related quality of life that captures both quality and quantity of time spent in a particular health state [73]. The EQ-5D-5L captures 243 unique health states based on the following domains: mobility, self-care, usual activities, pain, anxiety, and depression. A score of zero indicates death while a score of 1 indicates full health. The EQ-5D-5L also consists of a visual analog scale that records the individual’s self-related health on a vertical visual analog scale.

We will evaluate wellbeing using the ICEpop CAPability Measure for Older adults [74, 75]. This assesses wellbeing across five attributes: (1) attachment (love and friendship); (2) security (thinking about the future without concern); (3) role (doing things that make you feel valued); (4) enjoyment (enjoyment and pleasure); and (5) control (independence). A score of zero indicates no capability while a score of 1 indicates full capability.

##### Physical function

Cardiorespiratory fitness will be estimated with the modified Balke submaximal-graded exercise treadmill test [76], with heart rate continuously monitored. Participants will walk on the treadmill at a pre-determined speed (females 4.8 km/h; males 5.3 km/h) until they reach volitional fatigue or 70% of heart rate reserve (HRR). The Short Physical Performance Battery will assess general balance and mobility [77]. Participants are assessed on performance of standing balance, walking, and sit-to-stand. Each component is rated out of 4 points, for a maximum of 12 points. A score of < 9/12 predicts subsequent disability [77]. Participants will also complete the 400-m Walk [78]. Participants will be asked to walk 400 m as quickly as they can without running and time to complete will be recorded.

Muscular strength will be examined separately for the upper and lower body. Upper body strength will be assessed using the bilateral hand dynamometer grip strength test. Lower body strength will be assessed using the Biodex bilateral concentric knee extension and flexion strength test. The peak torque a participant is able to achieve will be recorded for each leg for both concentric extension and flexion. Additionally, lower body strength will be measured with the 30-s sit-to-stand test [79]; participants will be asked to complete as many full sit-to-stands as possible within 30 s.

Body composition will be assessed with a full-body dual-energy X-ray absorptiometry (DEXA) (Hologic, Mississauga, ON, Canada) scan. We will use DEXA-derived total body fat mass (g), total body lean mass (g), and body composition (% of fat and lean mass).

##### Actigraphy measures

Objective measures of sleep quality will be estimated over a 7-day period using the MotionWatch8© wrist-worn actigraphy unit (CamNtech; Cambridge, UK) to estimate sleep duration, latency, and fragmentation. Participants will also be asked to complete the Consensus Sleep Diary each morning [80]. In addition to sleep quality, the MotionWatch8© will also be used to calculate daily physical activity. The number of minutes spent in moderate to vigorous physical activity (>3.0 METs) is compared to the total time spent awake and out of bed to determine the percentage of waking hours each day spent in moderate to vigorous intensity physical activity [81].

##### Questionnaires and falls

Standardized questionnaires will be administered to assess anxiety [82], depression [83], sleep quality [84], sleep apnea risk [85], life space mobility [86], everyday memory [87], mindfulness [88], physical activity [89], sedentary behavior [90], social network [91], social provisions [92], loneliness [93], and health care costs [94]. Falls will be prospectively monitored via monthly calendars. See Table 1 for more details.

**Table 1:** Psychosocial assessments and other questionnaires

Name	Description
State and Trait Anxiety Inventory (STAI) [82]	A 40-item questionnaire assessing mood and anxiety.
Center for Epidemiologic Studies Depression Scale (CES-D) [83]	A 20-item questionnaire measuring symptoms associated with depression experienced in the past week.
Pittsburgh Sleep Quality Index (PSQI) [84]	A 19-item questionnaire assessing sleep quality in the previous month using subjective ratings for 7 different components (i.e., sleep quality; sleep latency; sleep duration; habitual sleep efficiency; sleep disturbance; use of sleeping medication; and daytime dysfunction).
STOP Bang Questionnaire [85]	An 8-item questionnaire assessing obstructive sleep apnea risk.
Life Space Questionnaire [86]	A 6-item questionnaire to measure the extent of mobility of older adults.
Everyday Memory Questionnaire (EMQ) [87]	A 28-item questionnaire assessing memory failures over the past 3 months.
Mindfulness Attention Awareness Scale (MAAS) [88]	A 12-item questionnaire assessing mindfulness during daily activities such as conversation, commuting, and eating.
CHAMPS Physical Activity Questionnaire for Older Adults [89]	A 41-item questionnaire assessing weekly frequency and duration of physical activities relevant for older adults.
Sedentary Behaviour Questionnaire (SBQ) [90]	A questionnaire assessing time spent in 9 sedentary behaviors during a weekday and weekend.
Lubben Social Network Scale – Revised (LSNS-R) [91]	A 12-item questionnaire assessing social engagement with family and friends.
Social Provisions Scale (SPS) [92]	A 24-item scale measuring the availability of social support.
UCLA Loneliness Scale [93]	A 20-item scale assessing feelings of loneliness and social isolation.
Health Care Resource Utilization (HRU) [94]	A 10-item questionnaire that asks participants about health care visits, services, and ability to do chores for the calculation of economic burden.

#### Tertiary outcomes

##### Brain structure and function

In a subset of participants at baseline and trial completion at 6 months, MRI will be conducted at the UBC MRI Research Centre using a Philips Achieva 3.0T MRI scanner with a 32-channel sensitivity encoding head coil (SENSE factor=2.4) and parallel imaging. High-resolution structural imaging will include two three-dimensional 1-mm isotropic T1 MPRAGE (TR=2530 ms, TE=3.04 ms, TI=800 ms, flip angle=10°, FOV=256×256×220 mm) that will be averaged during post-processing and two 1-mm isotropic T2 (SPACE) images (TR=4000 ms, TE=406 ms, flip angle=90°, FOV=260×228×176 mm). T1 images will be used for co-registration of individual participant functional MRI (*f*MRI) data into a standard stereotaxic space and for ascertaining changes in cortical thickness, and cortical and sub-cortical brain volumes. To allow the assessment of white matter hyperintensities (WMHs), we will acquire two sequences of T2-FLAIR. An Automated Labeling Pathway [95] will be used to quantify volumes and localization of focal WMHs. We will also use a 60-directional diffusion tensor image with a single shot echo-planar imaging (EPI) sequence (TR=7013 ms, TE=60 ms, FOV=224×224 mm, 70 slices, 2.2-mm slice thickness, voxel dimension=2.23 mm, *b*=700 s/mm2) and 5 non-weighted diffusion weighted images (*b*=0), with a focus on measuring fractional anisotropy, mean diffusivity, axial diffusion, and radial diffusion [96]. Sixty-direction acquisition will also generate the needed resolution for fiber-tracking. Functional network organization will be evaluated with resting-state functional MRI. EPI data will be acquired with slices in an oblique 30° orientation relative to main magnetic field to optimize signal in ventral frontal regions (TR=2000 ms, TE=30 ms, flip angle=90°, FOV=220×220 mm, 3.4×3.4×3.5 mm^2^ voxels). We will collect an additional 13-min scan to maximize test-retest reliability [97]. During resting state scans, participants will be instructed to keep their eyes open, look at a fixed point on a screen, and remain awake. We will also collect a letter n-back working memory task-evoked EPI scan as well as Relational and Item Specific Encoding and Recognition task-evoked EPI scans [98]. Preprocessing of the *f*MRI data will entail rigid body motion correction, spatial smoothing, band-pass temporal filtering to exclude confounding physiological signals, followed by an Independent Component Analysis based Automatic Removal of Motion Artifacts to remove motion-related artifacts. Preprocessed resting-state time-series will be extracted from a priori selected networks of interest masks based on the Yeo 7+ Networks [99] or parcellated regions of interest based on established functional atlases and cross-correlated with every voxel in the brain to establish functional connectivity maps. For preprocessed task-based *f*MRI data, within- and between-subject differences in task-evoked neural activity will be calculated via fixed effects and mixed effects modelling.

##### Cardiometabolic risk factors

We will assess arterial stiffness, blood pressure, body mass index, and fasting serum glucose and lipid profile. Arterial stiffness will be measured by carotid-femoral pulse wave velocity. Pulse wave velocity will be measured using a Complior DE1210 ultrasound machine (Alam Medical, Saint Quentin Fallavier, France) with the participants lying supine and rested for at least 5 min. Resting systolic and diastolic blood pressure will be recorded in duplicate, using a voscillometric sphygmomanometer, the Omron HEM-775. Values will be presented as a mean of two recordings that are taken 1 min apart. Participants will rest seated with their back supported, feet flat on the floor, and arms supported at heart level for 5 min prior to blood pressure measurements. Body mass index will be calculated as mass in kg/height in m^2^. Fasting serum glucose and lipid profile (e.g., total serum cholesterol, triglycerides, high-density lipoprotein, low-density lipoprotein, HbA1C) will be measured by conventional laboratory methods.

##### Hypothalamic-pituitary-adrenal axis function

To assess hypothalamic-pituitary-adrenal axis activity, we will measure the stress hormone cortisol to examine (1) total cortisol concentration over the day (area under the curve); (2) cortisol awakening response, a distinct aspect of the circadian cortisol profile; and (3) changes in cortisol response to engaging in an exercise session with time of day held constant. Free cortisol levels will be assessed in salivary samples (Salivettes) collected 5× each day (at awakening, +30 min, 2 pm, 4 pm, bedtime) for 2 days at baseline and trial completion at 6 months. As well, salivary samples will be collected immediately before and immediately after the exercise session within the first 2 weeks of exercise training and within the last 2 weeks of exercise training to examine cortisol response to exercise.

##### Blood biomarkers

Fasting blood will be collected in the morning at baseline and at trial completion at 6 months from all consenting participants. Blood will be processed and stored at −80 °C as plasma, serum, and whole blood in a secure research facility. The main analytes of interest include neurotrophic factors, myokines, sex steroid hormones, and pro- and anti-inflammatory cytokines. Remaining blood samples will be stored for 5 years from collection. If there are remaining samples after 5 years and are felt to be still useful for other studies, a separate ethics application will be submitted to explain the nature of its use. Otherwise, the samples will be destroyed after 5 years from collection.

### Treatment allocation

#### Randomization

After the baseline assessment, participants will be randomly assigned (1:1:1:1) to one of four experimental groups (Fig. 1). Randomization will be performed using the “randomizeR” package in R (Uschner et al. 2018). The randomization sequence will be stratified by biological sex with equal probability (1:1:1:1) random assignment to one of the four experimental groups: (1) 4×/week balance and tone (BAT; i.e., active control); (2) combined 2×/week AT + 2×/week RT (AT+RT); (3) 2×/week AT + 2×/week BAT (AT); or (4) 2×/week RT + 2×/week BAT (RT). Permuted blocks of size 12 will be used to ensure balance. To ensure blinding, the randomization sequence will be generated away from study personnel and held in a password-protected file on a secure university server and accessed only by a member of research staff who is not involved in this study.

#### Allocation concealment

Participant recruitment and enrollment will be managed by research personnel who will screen for eligibility, acquire consent, and conduct baseline assessments. Randomization to an intervention group will occur after completion of the baseline assessments. Research personnel conducting assessments and analyses will be blinded to group allocation. We will not be able to blind participants or research personnel delivering the interventions and obtaining the monthly physical activity questionnaire data. Blinding will be supported by providing explicit instructions to the research personnel and participants not to discuss issues related to physical activity during the assessments.

### Experimental groups

All participants will attend four 60-min sessions each week for 6 months (i.e., 26 weeks). All sessions will have a participant to instructor ratio of 4:1, with a maximum of 12 participants per session and will be led by certified exercise instructors. Each AT, RT, or BAT session will include a 10-min warm-up, 40min training, and a 10-min cool down. To ensure fidelity across different instructors and across time, detailed protocols with pictures will be provided during instructor training. Sessions will be regularly audited by the study coordinator with standard checklists to ensure intervention content is delivered accurately and consistently. For each AT, RT, or BAT session, exercise instructors will record attendance and participation (i.e., did participant complete all components of a given session). Borg’s Rating of Perceived Exertion (RPE) [100] will be monitored at standard intervals throughout each AT, RT, and BAT session.

#### AT program

The progressive moderate-intensity AT program has been used in prior trials and improved cognitive function in older adults with MCI [101, 102]. The AT program involves a series of standardized exercise stations, including treadmills, stationary cycles, aerobic steppers, agility ladders, and non-contact boxing. Participants rotate through each station within the 40-min training duration with 1-min rest between stations. Participants will exercise initially at approximately 50% of their age specific target HRR and gradually progress to reach the target of 80% of HRR. Exercise intensity during AT sessions will be monitored via heart rate monitors and the 20-point RPE [100]. Participants will gradually progress to a target RPE of 16 to 17.

#### RT program

The RT program has been used in prior trials and improved cognitive function in older adults [102, 103]. It is designed to significantly improve muscular strength using pressurized air system and free weights to provide the training stimulus. Pressurized air system exercises will consist of triceps extension, seated row, latissimus dorsi pull downs, leg press, and hamstring curls. Free weighted exercises will consist of bicep curls, standing calf raises, wall squats, and wall push-ups.

The first 6 weeks of the RT program will focus on orienting participants with resistance training and familiarizing them with proper technique. The initial intensity of the training stimulus will be set to a weight where participants can complete 2 sets of 10–15 repetitions. Training intensity will then progress on a cyclic basis from 45 to 85% of predicted 1 repetition maximum (1RM) as determined at week 6 using an 8-repetition maximum (8RM) test. Every 4 weeks, the 8RM test is repeated and the cycle repeats.

#### BAT program

The BAT program has been used in prior exercise trials as an active control [102–104]. It is a low-intensity program consisting of stretching exercises, range of motion exercises, static balance exercises (e.g., tandem balance, single leg balance), and functional strength exercises (e.g., sit to stand). Other than bodyweight and the occasional use of light resistance bands (e.g., 2 lbs or less), no additional loading (e.g., hand weights) will be applied to any of the exercises. There is no evidence that these exercises improve cognitive function [102, 103], and this group will serve to control for known and unknown confounding variables.

### Intervention adherence

Session attendance will be recorded by the instructors and adherence will be defined as the percentage of the total sessions attended.

### Data and adverse event monitoring

A Data and Safety Monitoring Committee will be established by co-investigators who will be independent from the day-to-day conduct of the study. They will review all adverse events on a quarterly basis and will classify them based on the definitions from the January 2007 OHRP *Guidance on Reviewing and Reporting Unanticipated Problems Involving Risks to Subjects or Others and Adverse Events, OHRP Guidance* (see link)**.** The Committee will stop the study if the data are of sufficient concern (e.g., increased rate of falls as a result of the intervention). All adverse events will be reported to this Committee and if required, to the relevant university and health authority ethics boards by the study coordinator. There is no anticipated harm or compensation for trial participation.

### Statistical analyses

Initial descriptive analyses will compute percentages, mean, or median values for baseline characteristics for each of the treatment arms. Next, missing data will be examined to identify the nature of missing data. Even if data are missing completely at random, missing data will be imputed to improve statistical power using multivariate imputation by chained equations [105]. Forty imputed data sets will be created following 40 iterations of a Gibbs sampler for each imputed data set. Proper convergence of the Gibbs sampler will be confirmed by visual inspection of trace plots of each imputed variable, to ensure proper mixing and the absence of spikes or systematic trends across iterations. Analyses will be pooled across the 40 imputed data sets.

The primary analysis will be performed according to the intention-to-treat principle and entail mixed effects ANCOVA to test the main effects of AT and RT, as well as the interaction effect of AT × RT (see Fig. 2) on changes in cognitive function, as assessed by the ADAS-Cog-Plus. The ANCOVA model will use the repeatedly measured (6-month and 18-month) ADAS-Cog-Plus performance as the dependent variable; baseline ADAS-Cog-Plus score, biological sex, AT, RT, and AT × RT as independent, fixed effects variables. Furthermore, to model changes in the outcome between months 6 and 18, and to estimate distinct treatment effects at months 6 and 18, a fixed main effect of time and all interactions between time and the independent variables will be included. To account for the dependency in the repeatedly measured outcome, a random subject-level intercept will be included. Month 6 will be considered the primary endpoint and month 18 will be considered the secondary endpoint. Secondary analyses of the primary outcome will examine the interaction between biological sex and effects of AT, RT, and AT × RT. A two-tailed alpha of 0.05 will be used for each comparison (i.e., uncorrected alpha).

Secondary outcomes will be examined using identical random-effects ANCOVA models as those constructed with the primary outcome.

#### Mediation and moderation

Mediation analysis will test for potential mediators of the relationship between the primary effects of exercise training (i.e., AT, RT, and AT × RT) and the outcome of interests (e.g., ADAS-Cog-Plus) [106]. The mediation effect will be quantified by the product of (1) the estimated effect of exercise training on the mediator, and (2) the estimated effect of the mediator on the outcome of interest. This product and its 95% confidence interval will be estimated using bootstrapping of the mediation model. To examine potential moderators (i.e., biological sex, genotype) of the relationship between exercise training and the outcome of interest, we will incorporate the potential moderator in the random-effects ANCOVA, including the main effect of the moderator and its interaction with the effects of AT, RT, and AT × RT.

#### Economic evaluation

The economic evaluation will examine the efficiency of three exercise interventions compared with BAT as well as ranking the relative cost-effectiveness of each of the three exercise interventions [107, 108]. The outcome of our cost–utility analysis is the incremental cost–utility ratio (ICUR): ICUR = ΔCost/ΔQALYs; QALYs, estimated from the EQ-5D-5L, represent time and quality spent in given health states.

### Modifications due to Covid-19 pandemic

In response to the COVID-19 pandemic and the restrictions put in place by the British Columbia government and UBC, all in-person research activities were halted on March 19, 2020. In-person research and enrollment of new participants were allowed to gradually resume as of August 11, 2020. In-person exercise sessions resumed at a limited capacity on September 28, 2020.

During this period of time, modifications were made to the protocol to allow the research team to continue the trial with enrolled participants. The modifications were done in two phases. Phase 1 modifications were implemented from March 19, 2020, until June 1, 2020. Phase 2 modifications were implemented from June 1, 2020, until August 11, 2020, when we resumed in-person assessments, or until September 28, 2020, when we resumed in-person exercise sessions. These modifications included the following:

(i) In-person, group-based exercise sessions were transformed into home-based exercise programs. Participants were provided with detailed exercise manuals with pictures and exercise calendars. The exercise calendars were used to track adherence as well as to record exercise intensity, using the Borg’s RPE [100]. For Phase 1, participants in the RT and AT+RT groups were given TheraBands and no other equipment was given. For Phase 2, the home-based exercise programs were augmented with YouTube exercise videos. For those without internet access, DVD’s were made and provided. In addition, RT participants were provided with weighted resistance bands, AT participants were given Fitbit Inspire HR (USA) fitness trackers to monitor heart rate during AT sessions, and BAT participants were given yoga balls. Weekly phone calls were made to answer any questions as well as to monitor any adverse events and falls. In Phase 2, exercise logs were provided to all participants. For RT, the weight used for each exercise, the number of reps and sets, and the RPE after each RT exercise were recorded. For AT, RPE as well as Fitbit measured heart rate were recorded at the beginning, in the middle, and at the end of each AT session. For BAT, the overall RPE was recorded per session.
(ii) All participants were contacted by phone twice per week. During these phone calls, instructors answered any questions, promoted adherence, and progressed RT and AT exercises when appropriate. Adverse events and falls were also tracked during these phone calls.
(iii) A total of 28 participants completed their intervention prior to August 11, 2020. To conduct their assessments, the research team revised the protocol to allow virtual measurement, where possible. We preloaded iPads with Zoom and delivered it with assessment packages (with instructions) to participants’ homes. Prior to implementation, the research team practiced and refined the virtual measurement protocol. All cognitive assessments were completed except for the (a) National Institutes of Health Toolbox List Sorting Working Memory Test; (b) spatial memory computerized test; (c) Rey-Osterrieth Complex Figure Test, and (d) Prospective Memory Test. For physical function measures, we measured the Short Physical Performance Battery, 400-m Walk, and 30-s sit-to-stand. We also provided a blood pressure monitor and had participants measure their blood pressure and heart rate while on Zoom. A total of 8 participants completed both their cognitive and physical assessments via Zoom. Another 5 participants completed their cognitive assessment in person but their physical assessment via Zoom. From June 24, 2020, to August 11, 2020, we received permission to conduct a limited number of in-person assessments. A total of 15 participants were assessed with appropriate social distancing and personal protective equipment being used. All cognitive assessments and a select number of physical assessments were completed.
(iv) Blood draws were not completed from June 1, 2020, to August 11, 2020. Saliva and MW8 data collection continued without any modifications.

Our 3T MRI Research Centre remained open throughout the pandemic with extra safety measures implemented, and thus, neuroimaging continued as long as participants were comfortable to do so.

On August 11, 2020, in-person assessments and blood draws resumed with approved safety protocols. On September 28, 2020, in-person exercise sessions resumed with approved safety protocols, including restrictions on occupancy. As AT and RT are of moderate intensity, to reduce the risk of COVID-19 among our participants while trying to preserve training fidelity, from August 11, 2020, to April 4, 2022, the four experimental groups were delivered using a hybrid model. In this hybrid model, participants completed two exercise sessions in person and completed two sessions at home each week. Specifically, the AT group completed AT sessions in person and BAT sessions at home. The RT group completed RT sessions in person and BAT sessions at home. The AT+RT group completed one AT and one RT session in person, and one AT session and one RT session at home. The BAT group completed two BAT sessions in person and two BAT sessions at home. Participants were provided with exercise manuals, YouTube videos, and exercise equipment. Exercise equipment included Fitbit Inspire HR fitness trackers, steppers, and agility cones for AT and weighted resistance bands for RT. Yoga balls were provided for BAT. Exercise logs were audited bi-weekly by exercise instructors.

We will conduct additional statistical analyses to compare our primary outcome (ADAS-Cog-Plus) between the participants whose training was significantly modified due to the COVID-19 pandemic.

## Discussion

We have assembled a unified, transdisciplinary, and experienced team to use a multi-pronged approach to assess the individual effects of AT and RT, as well as the interaction effect of combining the two types of exercise training on cognitive function and determine the possible underlying biological mechanisms in older adults with MCI. We expect that at the end of the 6-month intervention, compared with BAT, both AT and RT will demonstrate improved performance on the ADAS-Cog-Plus, in specific cognitive domains (i.e., executive functions and episodic memory), and in health-related quality of life, with the greatest improvements seen in the combined AT and RT group. We also hypothesize that the effects of AT and RT will be moderated by biological sex and genotype with greater AT benefits seen in females and those without the BDNF Val66Met polymorphism and greater RT benefits seen in males and in BDNF Val66Met polymorphism carriers.

Our study has some limitations that should be taken into consideration. Given our selection criteria, we will include older adults with MCI due to Alzheimer’s disease, vascular cognitive impairment, or both. Thus, our sample will be heterogeneous; however, this will increase the generalizability of our results. Despite the rigorous cognitive assessments included in our study, to ultimately appreciate the role of exercise in dementia prevention, studies that follow dementia progression rates are needed. Our study will provide critical data for future intervention trials powered to assess dementia progression rates as their primary outcome. We were unable to assess the sample size requirements for each of our secondary or tertiary outcomes. Thus, some of these outcomes will be underpowered.

This 2×2 RCT addresses the problem of how to effectively minimize cognitive decline among older adults with MCI—a population at increased risk for dementia—with different types of exercise. Establishing the efficacy of different types and combinations of exercise training will advance our understanding of how to best prescribe exercise to attenuate cognitive decline. Ultimately, our study results will allow decision makers to leverage the financial support needed for readily accessible and tailored community-based interventions to promote cognitive health and combat dementia.

### Trial status

This protocol is version 10, updated September 16, 2021. Participant enrollment began on November 13, 2017, and recruitment is anticipated to be completed by June 2023. Any changes to the protocol will be documented by the principal investigator and all research personnel will be notified. The clinical trial registration will be amended for all updates to the protocol.

## Data Availability

Only investigators and research teams with ethical approval will have access to the final datasets. The informed consent documents and the datasets used and/or analyzed during the current study will be available from the corresponding author on reasonable request.

## Abbreviations

AT: Aerobic training
RT: Resistance training
RCT: Randomized controlled trial
MCI: Mild cognitive impairment
BDNF: Brain-derived neurotrophic factor
IGF-1: Insulin-like growth factor-1
ADAS-Cog-Plus: Alzheimer’s Disease Assessment Scale-Cognitive-Plus
PAR-Q Plus: Physical Activity Readiness Questionnaire for Everyone
MoCA: Montreal cognitive assessment
MMSE: Mini-Mental State Examination
GDS: Geriatric depression scale
UBC: University of British Columbia
MRI: Magnetic resonance imaging
ANCOVA: Analysis of covariance
SMD: Standardized mean difference
EQ-5D-5L: EuroQol-5 domain-5 level
QALY: Quality-adjusted life years
HRR: Heart rate reserve
DEXA: Dual-energy X-ray absorptiometry
WMH: White matter hyperintensities
fMRI: Functional magnetic resonance imaging
EPI: Echo-planar imaging
APOE: Apolipoprotein
BAT: Balance and tone
RPE: Rating of perceived exertion
RM: Repetition maximum
ICUR: Incremental cost–utility ratio
